# Resistant hypertension post-transsphenoidal surgery for pituitary Cushing’s disease, leading to a diagnosis of primary aldosteronism

**DOI:** 10.1530/EDM-24-0137

**Published:** 2025-02-21

**Authors:** Jack Lee, Maria Tomkins, Darran McDonald, Julie Martin-Grace, Claire Carthy, John Finnegan, Douglas Mulholland, Neal Dugal, Arnold D K Hill, Michael W O’Reilly, Mark Sherlock

**Affiliations:** ^1^ Academic Division of Endocrinology, Royal College of Surgeons in Ireland, Dublin, Ireland; ^2^ Department of Endocrinology, Beaumont Hospital, Dublin, Ireland; ^3^ Department of Interventional Radiology, Beaumont Hospital, Dublin, Ireland; ^4^ Department of Surgery, Beaumont Hospital, Dublin, Ireland

**Keywords:** cases, articles, adrenal, cardiovascular, rare diseases/syndromes

## Abstract

**Summary:**

We describe a case of a 42-year-old gentleman, 5 years post-transsphenoidal surgery (TSS) for pituitary-dependent Cushing’s disease, initially presenting with malignant hypertension. Despite an initial improvement in his blood pressure post-TSS, he was found to be persistently hypertensive on follow-up despite no clinical or biochemical evidence of recurrence of hypercortisolism. His blood pressure remained elevated despite five antihypertensive agents. His renin concentration was <5 mIU/L (9–103.5) and aldosterone concentration was 877 pmol/L (0–670). A subsequent CT of the adrenals showed a 1.2 cm left adrenal nodule. He was not suitable for adrenal vein sampling (AVS) at this time due to difficult-to-control hypertension. Biochemistry was difficult to interpret in the context of a multitude of interfering medications, which were necessary given his difficult-to-control hypertension and hypokalaemia. Once suitable, his initial AVS was unsuccessful due to failure to cannulate the right adrenal vein. He was given the further options of repeat AVS vs ^11^C-metomidate PET vs medical management of his blood pressure. He proceeded with a repeat AVS, with successful cannulation of both adrenal veins. This showed evidence of hyperaldosteronism on the left side, with a lateralisation index of 39.5 and a contralateral suppression index of 0.28. He proceeded with a robotic left adrenalectomy, leading to significant improvement in his blood pressure, dropping from a mean reading of 142/85 during daytime and 150/88 mmHg at nighttime on five antihypertensive agents to normotensive levels of 114/77 mmHg on two agents.

**Learning points:**

- It is important to consider a broad differential for uncontrolled hypertension.
- It must be considered that patients can present with multiple, isolated endocrinopathies.
- There are diagnostic challenges with primary aldosteronism, with medication regimens regularly effecting suitability of testing and interpretation of results.
- AVS can be a challenging procedure, leading to diagnostic challenges in the lateralisation of primary aldosteronism; however, it or another form of lateralisation is essential to guide management options.

## Background

Resistant hypertension is a common presentation in endocrine clinics, with a broad differential diagnosis. Primary aldosteronism (PA) is the most common cause of secondary hypertension. Prevalence rates of PA are hard to define, but range from a low estimate of 1.0% to a high estimate of 36% of patients with resistant hypertension (1). PA can be a difficult diagnosis to make, and localisation studies with AVS can be a challenging procedure, with varying cannulation success rates from centre to centre (2). It is important to have a specialist centre with a high throughput of cases to help maximise radiologist experience.

In patients with significant hypercortisolism, cortisol can act as a mineralocorticoid as high levels of cortisol can overwhelm the 11β-hydroxysteroid dehydrogenase 2 (11β-HSD2) enzyme, whose role is to convert active cortisol to inactive cortisone. 11β-HSD2 protects the mineralocorticoid receptor from illicit occupancy by cortisol (3).

Although PA has been reported alongside adrenal Cushing’s syndrome, it is rarer to see it in a patient following treatment for pituitary Cushing’s disease, with no clear relationship between the two.

## Case presentation

A 42-year-old gentleman, 5 years post-successful transsphenoidal surgery (TSS) for Cushing’s disease, was found to be persistently hypertensive, despite biochemical evidence of Cushing’s disease remission. He also experienced episodes of spontaneous hypokalaemia and symptoms suggestive of obstructive sleep apnoea. His current medication regimen included hydrocortisone 10 mg/5 mg and desmopressin acetate 0.2 mg daily, following TSS, and antihypertensives ramipril 10 mg, amlodipine 10 mg, bisoprolol 10 mg, spironolactone 100 mg and doxazosin 8 mg.

## Investigation

The patient was biochemically in remission from a Cushing’s disease perspective, evidenced by an overnight dexamethasone suppression test, with a cortisol level of <50 nmol/L. There was no recurrence of his pituitary adenoma on MRI.

His potassium was normal at 3.9 mmol/L (3.5–5.3); however, he had a history of hypokalaemia, with levels of 3.0 and 3.1 mmol/L (3.5–5.3) in the past on routine samples in the 2 years before this presentation. There were normal values of 3.9–4.5 mmol/L (3.5–5.3) between these samples. His renal function and calcium were normal.

His blood pressure was elevated at 160/80 mmHg, and this was supported by a 24 h ambulatory blood pressure monitor (ABPM), with a daytime average of 142/85 mmHg and a nighttime average of 150/88 mmHg.

His peak cortisol on a short synacthen test was 378 nmol/L. He remained on hydrocortisone replacement.

Plasma metanephrines were normal. His serum renin concentration was <5 mIU/L (9–103), with a serum aldosterone concentration of 877 pmol/L (0–670). This was difficult to interpret in light of his medication regimen at the time including a mineralocorticoid receptor antagonist and beta-blockade; however, the picture was suggestive of aldosterone hypersecretion despite the above agents.

**Figure 1:**
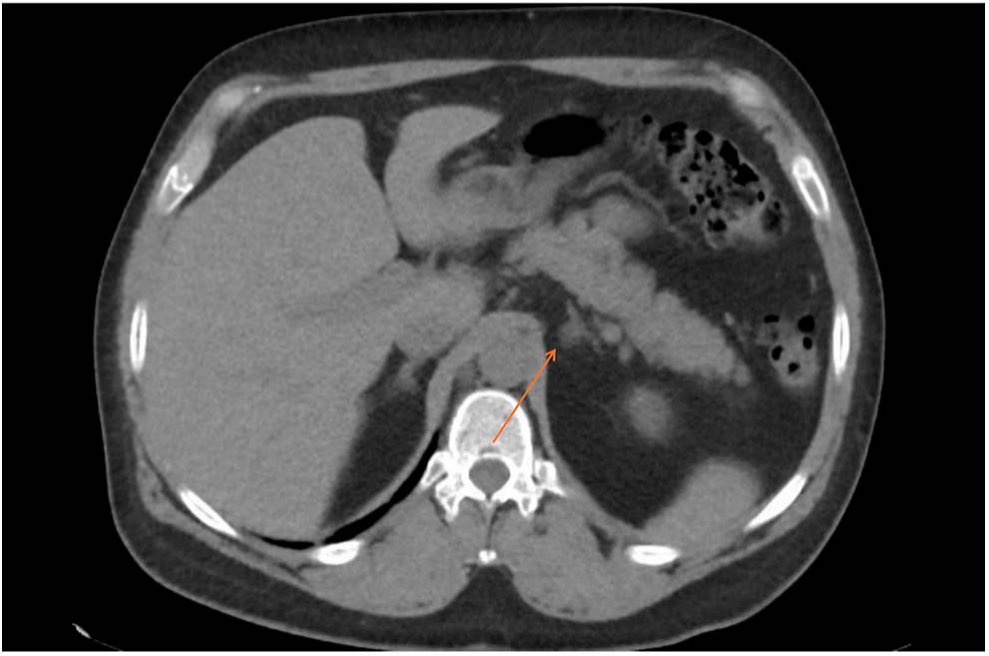
Axial view of CT of the adrenals showing a 1.2 cm left adrenal nodule of 35 houndsfield units.

**Figure 2:**
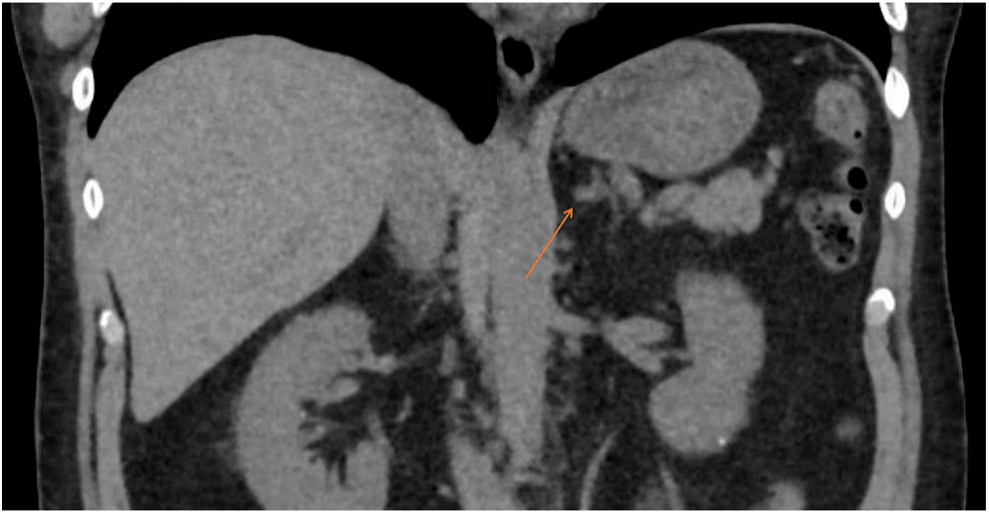
Coronal view of CT of the adrenals showing a 1.2 cm left adrenal nodule of 35 houndsfield units.

From an imaging perspective, he had a CT of the adrenals, showing a 1.2 cm left adrenal nodule of 35 houndsfield units (Figs 1 and 2). The right adrenal gland had a normal appearance. This remained stable on interval imaging throughout his further investigations.

The patient underwent AVS having transiently held his spironolactone with uptitration of doxazocin to 16 mg OD, with close monitoring of outpatient blood pressure and serum potassium.

On his initial AVS, unfortunately, the right adrenal vein could not be cannulated as evidenced by an adrenal vein: inferior vena cava cortisol (IVC) ratio <2. He had a repeat AVS with adequate cannulation of the right adrenal vein (right adrenal vein cortisol of 836 nmol/L, IVC cortisol of 219 nmol/L, ratio right adrenal vein: IVC cortisol = 3.6), with evidence of hyperaldosteronism lateralising to the left side (Table 1).

**Table 1:** Results of AVS.

AVS	Cortisol (C), nmol/L	Cortisol ratio*	Aldosterone (A), pmol/L	A:C ratio	A:C LAV/A:C RAV	A:C RAV/A:C IVC
IVC	231		816	3.5		
LAV1	5,929	25.6	147,860	24.9		
RAV1	836	3.6	525	0.63		
ALR					39.5	
CSI						0.18

AVS, adrenal vein sampling; IVC, inferior vena cava cortisol; ALP, aldosterone lateralization ratio (left-to-right); CSI, contralateral suppression index.

* AV:IVC (selectivity index).

## Treatment

Initially, during investigation, the patient was treated medically with uptitration of his antihypertensive agents to a regimen consisting of ramipril 10 mg OD, amlodipine 10 mg OD, bisoprolol 10 mg OD, spironolactone 100 mg OD and doxazocin XL 8 mg OD.

Treatment options were considered following the initial unsuccessful AVS; however, given his age, difficult-to-control hypertension and strong patient preference, he underwent repeat AVS, which was successful, lateralising to the side of the adrenal adenoma.

He proceeded with a robotic left adrenalectomy, which confirmed a 9 mm adrenocortical nodule, with a Weiss score of 0/9. Due to his history of secondary adrenal insufficiency, he required stress dosing of his hydrocortisone peri- and postoperatively. This was in the form of a dose of 100 mg IV at induction, followed by a stress dose of 20 mg PO TDS for 5 days, before resuming his regular dose of 10 mg BD.

## Outcome and follow-up

The patient recovered well in the initial postoperative period. He had pre-existing ACTH deficiency, following TSS, and remains on hydrocortisone replacement for this. Six months postoperatively, his systolic blood pressure is 114/77 mmHg on two agents (amlodipine 5 mg and bisoprolol 2.5 mg). His potassium is 4.7 mmol/L (3.5–5.3), with a renin of 20.4 mIU/L (9–103) and aldosterone of <138 pmol/L (0–670). According to the primary aldosterone surgical outcomes score, he has had complete biochemical and partial clinical resolution of his PA (4).

## Discussion

The recurrence rate of pituitary Cushing’s disease has been shown to be approximately 38% (5). This is important to take into consideration on follow-up, particularly in the work up of hypertension, as in this case. However, alternative differential diagnoses need to be taken into consideration. In patients with moderate-to-severe resistant hypertension (with or without hypokalaemia) and in hypertension with incidentalomas, the prevalence of PA is high and requires appropriate screening and definitive tests to establish the diagnosis.

In patients behaving like PA with hypertension and hypokalaemia, particularly in our case with the history of Cushing’s disease, glucocorticoid excess has to be considered and excluded. This was investigated with an overnight dexamethasone suppression test, which was normal.

Once the diagnosis of mineralocorticoid excess is made, it is crucial to make a distinction between unilateral and bilateral aldosterone hypersecretion, as this distinction significantly alters treatment options. Adrenal CT plays a role in the work up of PA patients; however, smaller adenomas may be missed and non-functioning incidentalomas may be picked up. In one study using AVS based on CT findings alone, 42 patients (21.7%) would have been incorrectly excluded as candidates for adrenalectomy and 48 (24.7%) might have had unnecessary or inappropriate adrenalectomy. AVS is an essential diagnostic step to distinguish between unilateral and bilateral adrenal aldosterone hypersecretion (6). In our case, the hypersecretion did lateralise to the side of the adrenal nodule; however, this could not be said with confidence without AVS or an alternative such as a ^11^C-metomidate PET (7).

Unilateral adrenalectomy is curative in patients with an aldosterone-producing adenoma and unilateral adrenal hyperplasia, and there is strong evidence to suggest that lowering of BP and serum aldosterone levels improves cardiac and cerebrovascular outcomes (8). It is important to reduce both BP and serum aldosterone, as it has been shown that patients with PA have a higher rate of cardiovascular events, independent of blood pressure (9). Surgery has been associated with lower all-cause mortality and major adverse cardiac events than targeted medical therapy (10), highlighting the importance of appropriate and timely investigation and subsequent surgical intervention in a case like this, given the patients young age and difficult-to-control hypertension.

Overall, it is important to consider alternative pathologies and endocrinopathies in patients who already have one endocrine diagnosis being followed up in the endocrine clinic. Once the diagnosis of PA is made, AVS plays a crucial role in lateralisation and ensuring the correct management approach is utilised. This potential for surgical management can be curative, with significantly improved cardiac and cerebrovascular outcomes.

## Declaration of interest

The authors declare that there is no conflict of interest that could be perceived as prejudicing the impartiality of the work reported.

## Funding

This work did not receive any specific grant from any funding agency in the public, commercial or not-for-profit sector.

## Patient consent

Written informed consent for publication of their clinical details and/or clinical images was obtained from the patient.

## Author contribution statement

JL, MT, DMcD and MS were involved in writing and reviewing the manuscript. MT, JMG, CC, MO’R and MS were involved in the endocrine clinical management of the patient. JF and DM were involved in the radiological investigation and intervention. ND and AH were involved in the surgical management of the patient.
